# Number of Children and Telomere Length in Women: A Prospective, Longitudinal Evaluation

**DOI:** 10.1371/journal.pone.0146424

**Published:** 2016-01-05

**Authors:** Cindy K. Barha, Courtney W. Hanna, Katrina G. Salvante, Samantha L. Wilson, Wendy P. Robinson, Rachel M. Altman, Pablo A. Nepomnaschy

**Affiliations:** 1 Maternal and Child Health Laboratory, Faculty of Health Sciences, Simon Fraser University, 8888 University Drive, Burnaby, British Columbia, V5A 1S6, Canada; 2 Department of Medical Genetics, University of British Columbia, Vancouver, British Columbia, Canada; 3 Child and Family Research Institute, Vancouver, British Columbia, V6T 1Z4, Canada; 4 Human Evolutionary Studies Program, Simon Fraser University, 8888 University Drive, Burnaby, British Columbia, V5A 1S6, Canada; 5 Statistics and Actuarial Science, Simon Fraser University, 8888 University Drive, Burnaby, British Columbia, V5A 1S6, Canada; University of Turku, FINLAND

## Abstract

Life history theory (LHT) predicts a trade-off between reproductive effort and the pace of biological aging. Energy invested in reproduction is not available for tissue maintenance, thus having more offspring is expected to lead to accelerated senescence. Studies conducted in a variety of non-human species are consistent with this LHT prediction. Here we investigate the relationship between the number of surviving children born to a woman and telomere length (TL, a marker of cellular aging) over 13 years in a group of 75 Kaqchikel Mayan women. Contrary to LHT’s prediction, women who had fewer children exhibited shorter TLs than those who had more children (*p* = 0.045) after controlling for TL at the onset of the 13-year study period. An “ultimate” explanation for this apparently protective effect of having more children may lay with human’s cooperative-breeding strategy. In a number of socio-economic and cultural contexts, having more chilren appears to be linked to an increase in social support for mothers (e.g., allomaternal care). Higher social support, has been argued to reduce the costs of further reproduction. Lower reproductive costs may make more metabolic energy available for tissue maintenance, resulting in a slower pace of cellular aging. At a “proximate” level, mechanisms involved may include the actions of the gonadal steroid estradiol, which increases dramatically during pregnancy. Estradiol is known to protect TL from the effects of oxidative stress as well as increase telomerase activity, an enzyme that maintains TL. Future research should explore the potential role of social support as well as that of estradiol and other potential biological pathways in the trade-offs between reproductive effort and the pace of cellular aging within and among human as well as in non-human populations.

## Introduction

Aging is a complex process that involves the progressive deterioration of biological functions, which affects quality of life, morbidity and mortality [1]. Importantly, the pace of this process varies greatly among individuals [2]. This variation may be partially explained by inter-individual differences in energy allocation to tissue maintenance versus reproduction during the reproductive years of each organism [3]. As the amount of energy an organism can mobilize at any given time is finite, life history theory (LHT) postulates that an increase in metabolic energy allocated to reproduction should result in a reduction in the amount of energy available for tissue maintenance [4–6]. Poor tissue maintenance, in turn, is expected to lead to faster cellular degradation and aging [7].

Findings from various species across taxa are consistent with the LHT prediction that increased reproductive effort should be linked to accelerated aging and, consequently, shorter lifespans [8–13]. The mechanisms mediating this phenomenon have not yet been fully investigated, but an acceleration of telomere shortening, a natural consequence of cellular aging, could be involved [14] [15–18]. Telomeres are repetitive nucleotide sequences located at the ends of chromosomes that protect against chromosomal DNA damage. Limitations in the ability of the DNA replication machinery to copy to the ends of chromosomes during cell division result in telomere shortening [19, 20]. Telomeres are also shortened as a consequence of DNA damage induced by oxidative stress [21]. Telomere shortening is countered by a ribonucleoprotein called telomerase that can elongate telomeres, thereby protecting the chromosome ends [22]. There are, however, limits to the ability of telomerase to preserve telomere length (TL) and, once telomeres reach a critically short length, the cell becomes senescent, a state of irreversible growth arrest [23].

In line with LHT’s prediction, a link between reproductive effort and TL has been reported by studies focused on non-human species. For example, in Atlantic silversides (*Menidia menidia*) higher egg production was associated with accelerated telomere shortening [12]. A similar relationship has been observed in various bird species, such as common terns (*Sterna hirundo*), zebra finches (*Taeniopygia guttata*) and blue tits (*Cyanistes caeruleus*), where naturally larger and experimentally augmented brood sizes have been associated with shorter telomeres [15, 18, 24]. Only two studies, thus far, have directly explored the relationship between reproductive investment and TL in mammalian species. Consistent with LHT’s prediction, a study comparing multiparous versus nulliparous wild-caught house mice (*Mus musculus*) showed a positive association between parity and the pace of telomere shortening [25]. A study on mink (*Neovison vison*), however, did not yield significant associations between parity and TL [26]. To our knowledge, no studies have been conducted in humans to explore the trade-off between the number of offspring women produce and changes in TL.

Here we prospectively evaluated the relationship between number of offspring and change in TL across a 13-year period in a cohort of indigenous Kaqchikel Mayan women. In this population, number of offspring is high and varies remarkably among individuals, providing a good model to investigate a potential association between reproductive effort and the pace of cellular aging in humans. Improving our understanding of the factors that influence inter-individual differences in TL changes can provide useful information to help improve our management of wellbeing, morbidity and mortality during the aging process.

## Methods

### Study Population and Participants

The study sample was drawn from two neighbouring indigenous rural Kaqchikel Mayan communities located in the southwest highlands of Guatemala. All of the participants were Kaqchikel Maya with at least five generations of traceable ancestors and a high degree of endogamy, which reduces genetic variability among individuals in this population compared to that of industrialized settings. Lifestyle was relatively homogenous amongst the participants. Specifically, women had similar diets, levels of physical activity, education, and socio-economic status. This homogeneity in lifestyle reduces the potential effects of confounding factors, thereby increasing our statistical power to detect the relationships of interest.

Participants were originally recruited in the year 2000 for the Society, Environment and Reproduction (SER) study, a prospective, naturalistic, longitudinal study that focused on the relationship between “real life” daily stress and women’s reproductive function [27, 28]. Recruitment in 2000 was conducted using the following inclusion criteria: women had to be parous, cohabitating with a male partner and not using any form of chemical contraceptive method [27, 28]. Of the 107 original SER participants, 94 volunteered to partake in the current study. In 2013 (13 years after the onset of SER), those 94 women ranged in age from 29 to 53. None of the participants smoked.

### Procedures

#### Ethics

Data collection and analysis in 2000 were approved by the University of Michigan’s Institutional Review Board. Secondary analysis of data collected in 2000 was approved for this study by Simon Fraser University’s Ethics Review Board. Data collection and analysis in 2013 were approved by Simon Fraser University’s Ethics Review Board and the University of British Columbia Clinical Ethics Review Board. Informed consent was obtained orally from illiterate individuals and in written form from literate ones. In all cases the consent document was read in Kakchiquel Mayan by a female research assistant to each prospective participant and signed by the participants with a cross, finger print or name initials, according to their individual preferences. The University of Michigan’s Institutional Review Board approved the 2000 consent procedure, and Simon Fraser University’s Ethics Review Board approved the one used in 2013.

#### Demographic questionnaire

In 2013 participants were asked to complete a short verbal demographic interview. The interview was administered in Kaqchikel (the native local language) by trained, local, bilingual female research assistants, and all answers were recorded by hand. The demographic interview included questions about each participant’s age, age at her first birth, maternal parity, inter-birth intervals, offspring survival, family income, diet, alcohol consumption and smoking habits. This questionnaire was used to determine each participant’s reproductive life history. “Change in number of surviving offspring” was defined as the number of children alive in 2013 that were born between 2000 and 2013. “Total number of surviving offspring” was defined as the number of children born to a woman since the onset of her reproductive career who were alive at the end of our observation period in the year 2013. Of the 94 women who volunteered for the current study, 75 completed the 2013 demographic interview and provided biospecimens in both 2000 and 2013.

#### Saliva and buccal sampling

TL was assessed for each participant at two time points 13 years apart (2000 and 2013). This is a sample of convenience. In the year 2000 only salivary specimens were collected, while in 2013 all participants provided a buccal epithelial cell sample. Thus, TLs in 2000 were measured in salivary specimens, while TLs in 2013 were measured in buccal epithelial cells. Importantly, saliva and buccal samples both contain a large number of epithelial cells [29], and TL in different tissues within an individual are known to be highly correlated [30–34]. Salivary specimens were collected by passive drool, transported on ice to the field laboratory, aliquoted into 2ml cryo-vials and stored at -10°C within 4 hours of being collected. The specimens were then transported from the field site to our laboratory on dry ice where they were stored at -80°C until analysis. Buccal specimens were collected using a SK-1 Isohelix Buccal Swab (Cat. No: SK-1S) with a Isohelix Dri-Capsule (Cat. No: SGC-50) to ensure the long-term stability of buccal DNA on the swab head. Buccal specimens were collected according to the manufacturer’s instructions. Briefly, the sterile swab was removed from the tube, inserted into the mouth of the participant and rubbed firmly for 1 minute against the inside of the right cheek. The swab was then placed back into the tube along with a Dri-Capsule and the tube was closed and secured. Buccal samples were transported to the field laboratory and stored at room temperature in a dry, dark cabinet for approximately 2 months before being transported at room temperature from the field site to our laboratory where they were stored following the same storage protocol until analysis.

#### Telomere length assay

Genomic DNA was extracted from saliva and buccal samples. Saliva cells were washed with sterile PBS prior to a standard ethanol precipitation extraction using the Gentra PureGene Cell Kit (Qiagen, Hilden, Germany). Buccal samples were extracted with the DDK DNA Isolation Kit (Isohelix Ltd, Kent, UK), following the manufacturer’s instructions. All DNA samples were diluted to 10ng/uL in 10mM Tris, 0.1mM EDTA buffer. Average relative TL was measured using quantitative PCR (qPCR) as previously described [35]. Although measurement of TL by Southern blot of the terminal restriction fragment (TRF) is considered the ‘gold standard’ in the field, qPCR is also a widely used and accepted technique for assessing TL. qPCR is particularly useful in population-based studies, such as this one, because it requires much smaller amounts of DNA [36]. An advantage of the qPCR-based method is its high reproducibility [37, 38], which has resulted in a recent increase in the use of qPCR for TL measurement [36]. Briefly, SYBR green was used to quantify the amplification of telomeric repeats relative to the single-copy gene, *36B4*. The design of the telomere primers allows for amplification of the smallest possible amplicon (76bp); as a result, the qPCR amplification (C_T_ value) is proportional to the amount of primer binding sites in the genomic DNA template [39]. qPCR does require attentive preparation and stringent criteria to be reproducible due to higher inter-assay variation in comparison to other methods [37, 38]. For that reason, a stringent criteria was applied to our methodology: samples were run in triplicate in each 96-well plate, with at least two independent replicates. Furthermore, we used a rigorous threshold for the variation between replicates as C_T_ values with a standard deviation greater than 0.20 between triplicates were excluded. If the coefficient of variation between the two independent replicates was less than 0.15, the replicates were averaged to obtain a relative TL for each sample. For those samples that did not meet these criteria, two additional independent replicates were performed using the same quality control thresholds. Average relative TL for each sample was expressed as the ratio of the telomere repeat copy number to the single-copy gene copy number (T/S ratio). The T/S ratio has previously been shown to be proportional to the average TL of a given sample [39]. We have previously validated the reproducibility of our methods by calibrating and optimizing our qPCR methodology by blind comparison with sample measurements obtained by the flow FISH technique (r = 0.96; [35]).

### Statistical analysis

Two separate multiple regression models were used to evaluate the relationships between TL in 2013 and 1) “change in number of surviving offspring (2000–2013)”, maternal age at first birth and average inter-birth interval, as well as 2) “total number of surviving offspring”, maternal age at first birth and average inter-birth interval using JMP (version 12; SAS Institute). We tested for multicollinearity amongst the variables in each multiple regression model by examining each variable’s variance inflation factor (VIF) [40]. All VIF values were less than 10, indicating that these variables were not correlated with each other [40]. As TL in 2000 and 2013 were measured in different tissues, we decided to take a conservative approach and include salivary TL in 2000 as a covariate in our model to control for each participant’s initial TL in 2000 rather than to use it to calculate change in TL between 2000 and 2013. Saliva and buccal samples contain a large number of epithelial cells [29], and TL in different tissues within an individual are known to be highly correlated [30–34]. Women’s ages in 2013 were also included as a covariate in both models. Significance levels were set at α = 0.05.

## Results

### Descriptive statistics

The 75 participants included in these analyses were, on average, 19.8 years old at the birth of their first child (range = 14–33, SD = 3.1) and 39.4 years old in 2013 (range = 29–53, SD = 5.6) These women gave birth to an average of 5.6 surviving children in total (range = 1–10, SD = 2.1) and 2.9 surviving children between 2000 and 2013 (range = 0–6, SD = 1.5). Average inter-birth interval was 3.2 years (range = 1.9–6, SD = 1.0;). The average T/S ratio in saliva in 2000 was 2.75 (range = 1.38–7.72, SD = 1.03) and in buccal cells in 2013 it was 1.53 (range = 0.57–2.60, SD = 0.34). Descriptive statistics are summarized in Table 1.

**Table 1:** Descriptive study population statistics (n = 75).

Variables	Mean	Standard Deviation
Age in 2013 (years)	39.4	5.6
Salivary telomere length in 2000 (T/S ratio)	2.75	1.03
Buccal telomere length in 2013 (T/S ratio)	1.53	0.34
Change in number of surviving offspring from 2000 to 2013	2.9	1.5
Total number of surviving offspring	5.6	2.1
Maternal age at first birth (years)	19.8	3.1
Average inter-birth interval (years)	3.2	1.0

### Reproductive history and TL

**Fig 1:**
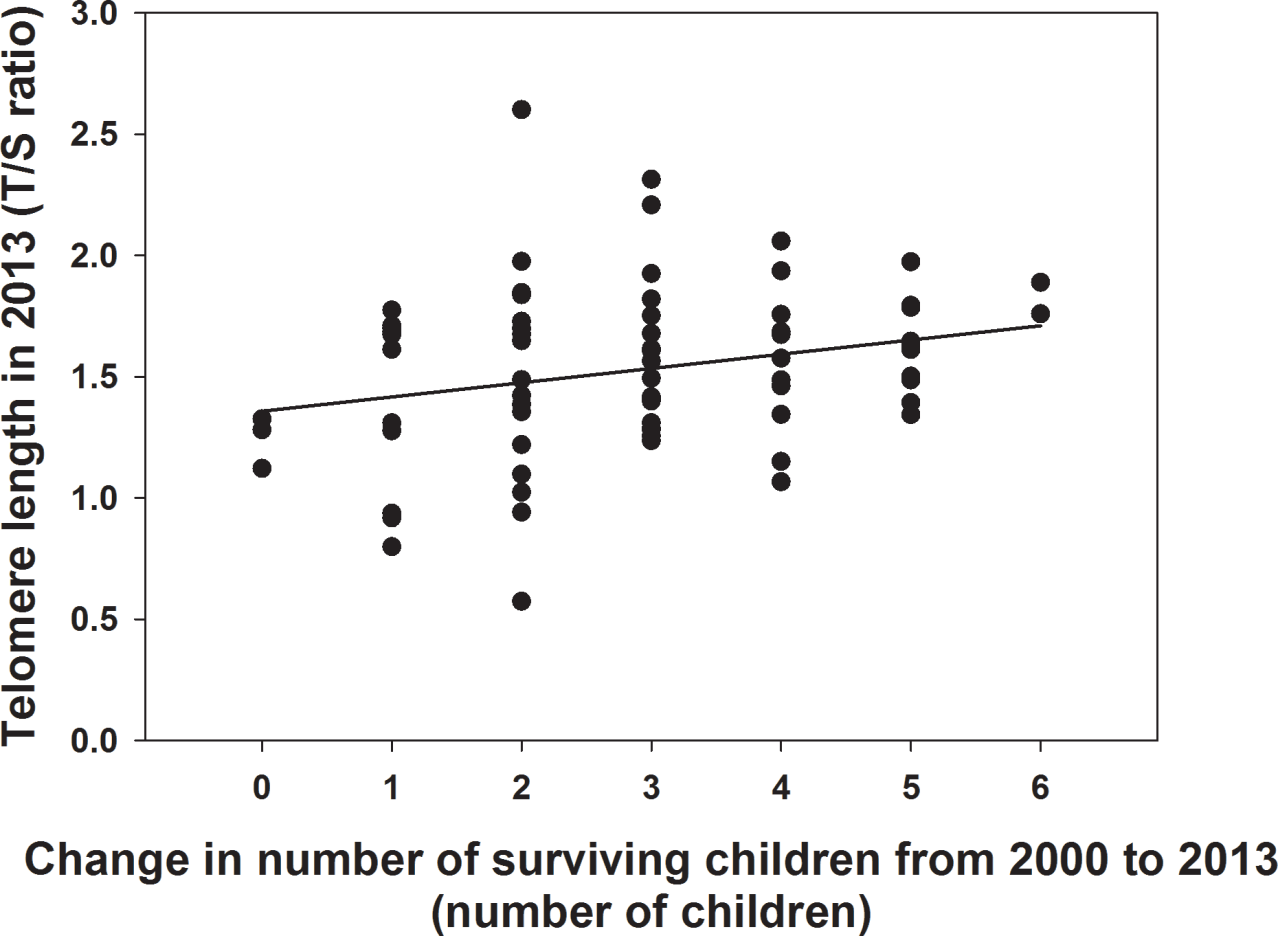
Change in number of surviving children and telomere length. Women who had more children from 2000 to 2013 had longer telomere lengths in 2013 than women who had fewer children, after controlling for their telomere length in 2000 and age in 2013.

After controlling for TL in 2000 and age in 2013, change in number of surviving offspring was positively associated with TL in 2013 [*p* = 0.029; Table 2, Model 1]. Specifically, each additional child born between 2000–2013 was associated with 0.059 more TL units in his or her mother (Fig 1). After controlling for TL in 2000 and age in 2013, a positive, yet marginally significant, association was observed between total number of surviving offspring (i.e., number of children born and surviving to a woman since the onset of her reproductive career until 2013) and TL in 2013 [*p* = 0.068; Table 2, Model 2]. Maternal age at first birth and average inter-birth interval were not significantly associated with TL in 2013 in either model [all *p* > 0.2; Table 2].

**Table 2:** Relationship between telomere length in 2013 and women’s reproductive history variables. **Model 1:** Change in number of surviving offspring from 2000 to 2013 was positively associated with telomere length in 2013, controlling for salivary telomere length in 2000 and maternal age in 2013 **(n = 75; adjusted R**^**2**^ **= 0.041). Model 2:** Total number of surviving offspring was marginally positively associated with telomere length in 2013, controlling for salivary telomere length in 2000 and maternal age in 2013 **(n = 75; adjusted R**^**2**^ **= 0.021).** Neither maternal age at first birth nor average inter-birth interval was associated with telomere length in 2013 in either model.

**Model 1**	**Estimate**	**Standard Error**	**t-ratio**	**p-value**
Intercept	1.240	0.439	2.82	0.0062
Age in 2013	0.001	0.008	0.15	0.8781
Salivary telomere length in 2000	0.049	0.040	1.23	0.2226
Change in number of surviving offspring from 2000 to 2013	0.066	0.029	2.23	0.0290
Maternal age at first birth	-0.013	0.013	-0.95	0.3449
Average inter-birth interval	0.051	0.042	1.21	0.2319
**Model 2**	**Estimate**	**Standard Error**	**t-ratio**	**p-value**
Intercept	1.507	0.399	3.78	0.0003
Age in 2013	-0.018	0.010	-1.75	0.0852
Salivary telomere length in 2000	0.050	0.041	1.23	0.2243
Total number of surviving offspring	0.052	0.028	1.86	0.0676
Maternal age at first birth	0.006	0.017	0.35	0.7250
Average inter-birth interval	0.057	0.044	1.29	0.2004

## Discussion

In contrast with LHT’s prediction that increased number of offspring should accelerate the pace of cellular aging, in our study population women who had more children during the 13-year observation period presented with longer TLs than those who had fewer children. A similar trend, albeit only marginally significant, was observed when the total number of surviving children born to a woman since the onset of her reproductive career was considered. These results suggest that, at least in our study population, having more surviving children acts as a protective factor, slowing the pace of telomere shortening. Mechanistically, this protective effect may be explained by the actions of the gonadal steroid estradiol, which increases dramatically during pregnancy (up to 200 times non-pregnancy levels) and is known to act as a TL protective factor. Indeed, estradiol is a potent antioxidant that reduces cellular exposure to oxidative stress [41–43]. Oxidative stress is known to lead to TL shortening [44]. Women who experience more pregnancies are exposed to higher estradiol levels, which indirectly protect cells from TL shortening by reducing exposure to oxidative stress. Moreover, estradiol also increases telomerase activity, an enzyme that maintains TL [45]. The protective effects of parity and estradiol have been supported by animal studies. Experimental manipulation of estradiol in mice resulted in larger litter sizes and was associated with increased levels of anti-oxidants and less oxidative damage [46, 47]. Future studies should explore a possible link between offspring number, gonadal steroid production and the pace of cellular aging in naturalistic conditions in humans and other mammalian species.

Importantly, to our knowledge, no other study has previously examined the direct association between number of children and longitudinal telomere length shortening in humans. In a cross-sectional analysis of leukocyte TL and age at natural menopause, Gray et al. (2014), included number of children as a covariate [48]. In contrast to our findings, they report a negative relationship between number of children and TL in their sample of 486 white post-menopausal women from the USA. The disparity between their results and ours may be explained by a number of methodologic and demographic differences between the two study populations. For example, Gray and colleagues looked at the relationship between TL and number of children retrospectively, while we did so prospectively, controlling for TL at the onset of our study period. It is possible that the negative association between number of children and TL that Gray et al. observed reflects initial differences in TL that existed among women before they began having children. In other words, the differences in TL they observed may not be related to differences in the number of children women in their study had [49]. The two study populations also differed in the participants’ reproductive status and age. While the participants in Gray et al. were, on average, 74.9 years old and all post-menopausal, our participants were, on average, 39.4 years of age at the end of our study period when TL was assessed (2013). This difference in age may be important because the rate of decline in TL is thought to be lower during women’s reproductive years than later in life [13, 50]. Indeed, by the time TL was assessed in the study by Gray and colleagues, the post-menopausal women had been without the protective effects of endogenous estrogens for over 25 years (average age at menopause in that study was 48.7 years). Exposures taking place in the life of those women over that 25-year post-reproductive period could conceivably mask the protective effects that children may have had at younger ages. Additionally, the two study populations differed dramatically in their ethnic background and socio-ecological (i.e., agricultural versus industrial) environment. Thus, the two groups of women were likely exposed to different diets, physical activity budgets, and social support systems, among other relevant factors, which, in turn, are likely to have differentially influenced the pace of TL shortening [50]. In sum, the differences in the results between our study and that of Gray and colleagues may be the consequence of differences in socio-ecological contexts between the two groups of women studied.

Indeed, socio-ecological differences between and within human populations may play an important role in the pace of TL shortening. A brief survey of the existing literature suggests that allomaternal care (i.e., care delivered by members of a woman’s social support network such as fathers, grandmothers, aunts, siblings, etc.), for example, may be a potential modulator of the relationship between reproductive effort and the pace of aging in women. Humans are cooperative breeders and, in some contexts, the number of children a woman bears may be directly related to the amount of social, logistic, and material support she receives. Thus, more children may lead to greater support, which in turn, may lead to an increase in the amount of metabolic energy that can be allocated to tissue maintenance, thereby, slowing the process of cellular aging. Consistent with this hypothesis, anthropological studies show that maternal support and allomaternal care decreases maternal energy expenditure associated with child rearing [51–55]. Previous studies have also shown that increased social support buffers against shortening of telomeres [50, 56]. Thus, we propose that the combination of support received by mothers in these societies may help them defray the costs of reproduction, thereby freeing metabolic energy, part of which could be allocated to tissue maintenance, thereby reducing the pace of cellular aging.

Future studies should formally test the relationship between the availability of maternal support, reproductive effort and the pace of cellular aging. Doing so will require a revision of the way reproductive effort is assessed in humans as allomaternal care affects the amount of energy required to produce viable offspring. Thus, the nature of the association between reproductive effort and the pace of cellular aging is likely to differ between species in which cooperative breeding strategies are used and those where these strategies do not exist. In the case of humans, we hypothesize that in societies with greater social support, women will show a slower pace of cellular aging than those living in societies with lower levels of social support. Similar relationships should also be expected in non-human species that engage in cooperative breeding.

It should be noted that while several studies have reported evidence linking TL and the aging process in ‘natural’ (non-laboratory) conditions for several species, including humans [57–61], TL is not yet universally accepted as a biomarker of aging [62]. TL is related to several age-associated variables and with mortality in humans, but not all studies find these associations [63–68]. Nonetheless, changes in TL are broadly accepted to be associated with the process of **cellular** aging, which is a known component of biological aging [69–71], albeit not the only one.

In summary, our analyses show that increased offspring number across 13-years of observation attenuated telomere shortening, suggesting that, in our study population, having more children may slow the pace of cellular aging. Although we do not directly measure TL in the same tissue longitudinally, our unique study design allowed us to correct for initial TL in each individual, overcoming the major limitation of cross-sectional studies [48]. Our results add to the growing literature examining the relationship between offspring number and aging. Importantly, in humans some studies finding positive associations between offspring number and longevity but others fail to find any relationships or find negative ones (for review see [72]). Together these mixed results emphasize the complexity of the relationship between offspring number and the pace of aging in women. Indeed, our regression model explained approximately 4% of the variation in TL shortening, and the proportion of variance explained by offspring number must be even smaller, suggesting that telomere dynamics are also likely affected by other important factors.

Although at first glance our results seem to contradict LHT’s prediction, they would, in fact, be consistent with it if our hypothesis regarding the role of cooperative breeding is supported by future studies. Reproduction involves the investment of energetic resources and increased health risks which may lead to lower investment in somatic effort. In humans and other cooperative-breading species, however, pregnancy and offspring rearing may attract higher social support resulting in a net energetic gain, which in turn may slow down the aging process. Larger longitudinal studies with women of different ethnicities as well as different social and physical environments are needed to test our hypothesis that “cooperative breeding” may modulate the relationship between reproductive effort and the pace of biological aging in humans as well as to explore other relevant socio-ecological and biological factors.
